# Pulmonary thromboembolism: a case report and misdiagnosis analysis of a 63-year-old female patient

**DOI:** 10.3389/fmed.2024.1411338

**Published:** 2024-08-13

**Authors:** Yingli Deng, Jing Lai, Qingmin He

**Affiliations:** ^1^Second Ward, Department of Respiratory and Critical Care Medicine, Ankang Central Hospital, Ankang, Shaanxi, China; ^2^Department of Gastroenterology, Ankang Central Hospital, Ankang, Shaanxi, China

**Keywords:** pulmonary thromboembolism, pneumonia, misdiagnosis, diagnosis, case report

## Abstract

This paper presents a case of a 63-year-old female patient who was initially misdiagnosed with mycoplasma pneumonia due to symptoms such as chest pain, hemoptysis, and fever, but was later confirmed to have pulmonary thromboembolism (PTE) through further examination. This case highlights the similarities between PTE and pneumonia in terms of symptoms, as well as the complexity of PTE diagnosis. The article provides a detailed description of the patient’s medical history, symptoms, examination process, and treatment outcomes. Furthermore, it discusses the possible reasons for the misdiagnosis, including insufficient awareness of PTE among physicians, lack of in-depth investigation into the causes of abnormally elevated D-dimer levels, the non-specific clinical manifestations of PTE, and the concerns of the patient’s family regarding pulmonary artery CTA examination. Additionally, the article emphasizes the importance of clinicians in improving their ability to differentiate and diagnose PTE, rationally utilizing clinical examination methods, and ensuring timely diagnosis and treatment of PTE.

## 1 Introduction

Pulmonary thromboembolism (PTE) is a disease triggered by thrombi originating from the venous system or right heart, which obstruct the pulmonary arteries or their branches, leading to impairments in pulmonary circulation and respiratory function (1, 2). As one of the common cardiopulmonary vascular diseases in China (3, 4), the primary clinical manifestations of PTE include dyspnea, chest pain, and cough. However, these symptoms closely resemble the respiratory symptoms of mycoplasma pneumonia, leading to frequent misdiagnosis and subsequent missed diagnosis, which can severely impact the prognosis of patients. This paper aims to illustrate a typical case of PTE that was misdiagnosed as pneumonia.

## 2 Case presentation

A 63-year-old female presented to our hospital with complaints of chest pain and hemoptysis for 18 days, accompanied by fever for 13 days. Her symptoms began while traveling on a long-distance train, manifesting as right-sided chest pain and mild cough with scanty dark red bloody sputum, which gradually worsened. Despite receiving symptomatic treatment (including cough suppression, analgesia, and fever reduction) at the local health clinic, her condition did not improve. Thirteen days prior to admission, she developed fever and was diagnosed with mycoplasma pneumonia, where she was treated with piperacillin, moxifloxacin, and other medications. Although the fever resolved, her chest pain and hemoptysis persisted, and she developed shortness of breath. A chest CT scan revealed an increase in lung lesions.

Upon admission to our hospital, her initial physical examination revealed a blood pressure of 135/75 mmHg, a pulse oximetry reading of 92%, and decreased breath sounds in the right lower lung with few wet rales audible. The remaining lung fields had slightly coarse breath sounds. There was pitting edema in both lower extremities, with no other significant abnormalities noted. Her preliminary diagnoses were: (1) Community-acquired pneumonia (non-severe), (2) Pleural effusion, (3) Pulmonary hemorrhage, and (4) Hypoxemia. Differential diagnoses: (1) Acute lung abscess; (2) Bronchiectasis with infection. She was initially treated with anti-infective therapy (Intravenous infusion of moxifloxacin 0.4 g qd for 1 week), oxygen inhalation, and other symptomatic measures.

**Figure 1:**
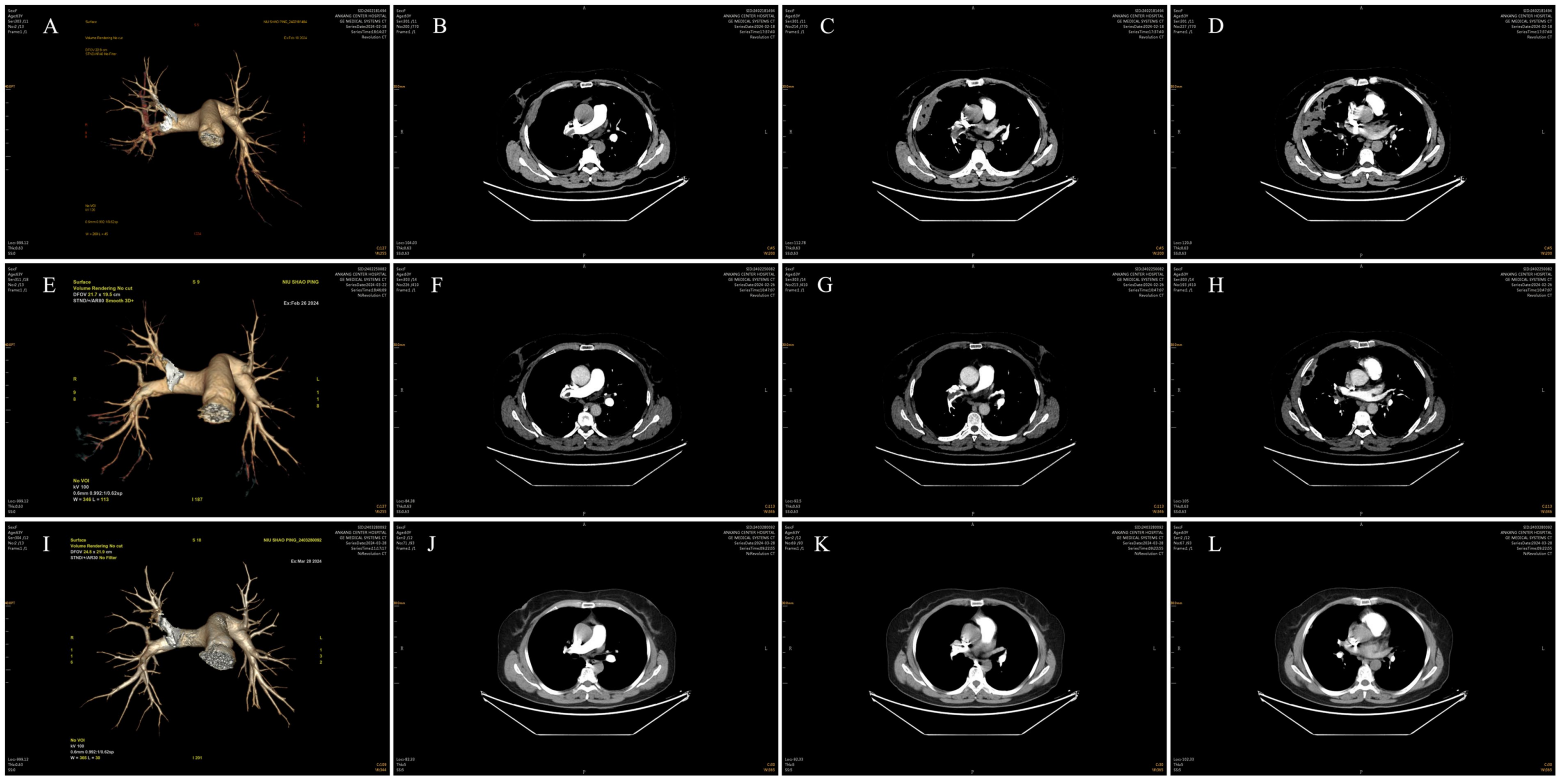
Pulmonary artery CTA. **(A–D)** Pulmonary artery CTA showed emboli in the main trunk of the right pulmonary artery and its branches in the upper, middle, and lower lobes of the right lung. **(E–H)** Pulmonary artery CTA demonstrated slight resorption of emboli in the main trunk of the right pulmonary artery and its branches in the upper, middle, and lower lobes of the right lung compared to previous images. **(I–L)** Pulmonary artery CTA showed a reduction in emboli in the main trunk of the right pulmonary artery and its branches in the upper, middle, and lower lobes of the right lung compared to previous images.

Subsequent investigations revealed sinus bradycardia and T-wave changes on ECG. Pulmonary artery CTA showed emboli in the main trunk of the right pulmonary artery and its branches in the upper, middle, and lower lobes of the right lung (Figures 1A–D). The scanned right lung appeared to have inflammatory changes, and partial pulmonary infarction could not be excluded. There were no significant abnormalities on bilateral lower extremity vascular ultrasonography. Echocardiography revealed mild tricuspid regurgitation and reduced left ventricular diastolic function. Blood gas analysis indicated an oxygen partial pressure of 67.00 mmHg at a 35% oxygen inhalation concentration, with a calculated oxygenation index <200 mmHg, suggesting type I respiratory failure. Coagulation studies revealed a fibrinogen concentration of 5.36 g/L and a D-dimer level of 10.80 mg/L. Blood tests showed a C-reactive protein level of 22.71 mg/L, a hypersensitive C-reactive protein level > 10.0 mg/L, and a procalcitonin level of 0.760 ng/mL. Both sputum TN-RNA and general bacterial cultures were negative. Chest ultrasonography detected a small amount of fluid in the right costophrenic angle.

Based on the aforementioned auxiliary examinations, the revised diagnoses are as follows: (1) Pulmonary embolism (submassive), (2) Type I respiratory failure, (3) Pulmonary infarction, (4) Pulmonary hemorrhage, (5) Community-acquired pneumonia (non-severe), (6) Mycoplasma infection, (7) Exudative pleural effusion, (8) Sinus bradycardia, and (9) Hyperfibrinogenemia. An anticoagulation regimen was initiated with subcutaneous injections of heparin calcium 5,000 IU twice daily for 5 days, concomitant with anti-infection therapy, oxygen inhalation, and symptomatic treatment. Following improvement in hemoptysis, oral rivaroxaban 15 mg twice daily was administered as sequential therapy, with no changes to the remainder of the treatment plan. After undergoing the aforementioned therapies, the patient exhibited significant alleviation of chest pain, hemoptysis, and shortness of breath, with no recurrence of fever.

Subsequent investigations were conducted to further evaluate the patient’s condition. Sputum pathology revealed no malignant tumor cells. Dynamic electrocardiogram showed sinus bradycardia with occasional atrial and ventricular premature beats, while ST-T segments remained unremarkable. Pulmonary artery CTA demonstrated slight resorption of emboli in the main trunk of the right pulmonary artery and its branches in the upper, middle, and lower lobes of the right lung compared to previous images (Figures 1E–H). Additionally, the scanned lesions in the right lung showed slight resorption, suggestive of inflammation, although partial pulmonary infarction in the right lung could not be excluded. Repeat blood gas analysis was essentially normal, and D-dimer levels decreased significantly.

The discharge diagnoses encompassed the following: (1) Pulmonary embolism (submassive), (2) Type I respiratory failure, (3) Pulmonary infarction, (4) Pulmonary hemorrhage, (5) Community-acquired pneumonia (non-severe), (6) Mycoplasma infection, (7) Exudative pleural effusion, (8) Sinus bradycardia, (9) Hyperfibrinogenemia, (10) Atrial premature beats, and (11) Ventricular premature beats.

**Figure 2:**
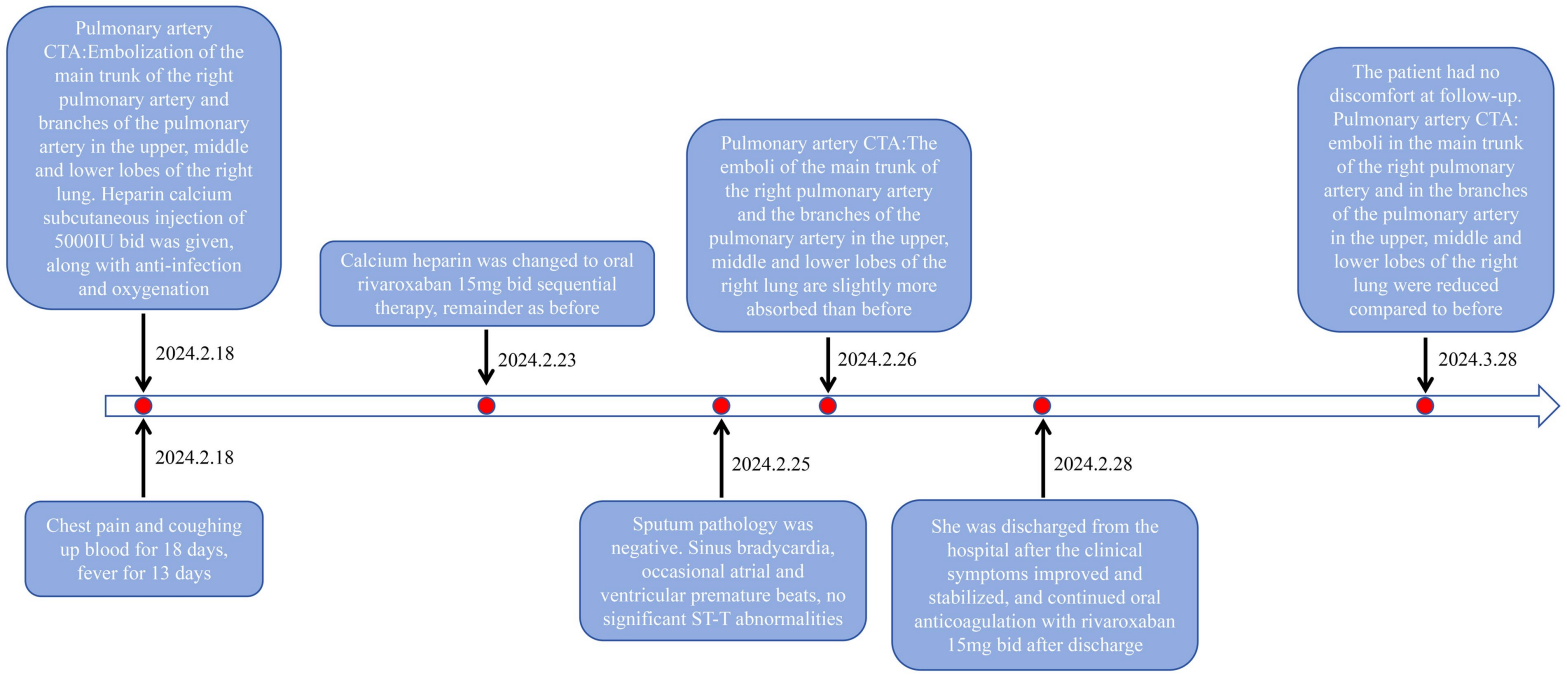
Treatment history of the case.

Following clinical improvement and stabilization after anticoagulation, anti-infection therapy, oxygen therapy, and other treatments, the patient requested discharge. Oral anticoagulation therapy with rivaroxaban was continued after discharge. One month later, during a follow-up visit, the patient reported no chest pain, dyspnea, or hemoptysis. Repeat pulmonary artery CTA showed a reduction in emboli in the main trunk of the right pulmonary artery and its branches in the upper, middle, and lower lobes of the right lung compared to previous images (Figures 1I–L). Moreover, the scanned lesions in the right lung had decreased in size. The detailed illustration of the entire treatment process is provided (Figure 2).

## 3 Discussion

PTE, the most common clinical type of pulmonary embolism, primarily results from the obstruction of pulmonary arteries and their branches by emboli formed due to the detachment of deep venous thrombi in the lower extremities. This blockage subsequently leads to a clinical syndrome caused by the interruption of tissue blood supply. In recent years, the incidence of PTE among the elderly population has been increasing year by year. According to relevant reports, the inpatient mortality rate of elderly PTE patients aged 65 and above is 3–10 times higher compared to younger patients, posing a severe threat to the health of the elderly.

The classic triad of symptoms in pulmonary infarction includes dyspnea, chest pain, and hemoptysis. However, these symptoms do not always occur simultaneously in patients with pulmonary infarction. Additionally, the nonspecific nature of their clinical manifestations often leads to delays in diagnosis. To facilitate more effective diagnosis of PTE, the following clinical examination methods may provide valuable assistance.

1. Pulmonary Embolism Rule-Out Criteria (PERC): The PERC score serves as a tool for assessing the risk of pulmonary embolism based on a series of clinical factors, encompassing patient age, oxygen saturation (SaO2), pulse rate, hemoptysis, recent trauma or surgery history, venous thromboembolism (VTE) history, unilateral leg swelling, and a history of oral steroid use (5). Specifically, a patient with an age under 50, absence of hemoptysis and unilateral leg swelling, pulse rate below 100 beats per minute, oxygen saturation above 94%, and no history of VTE, recent trauma or surgery, or oral steroid use, is considered to have a negative PERC score, indicating a relatively low risk of pulmonary embolism. Conversely, the presence of any one or more positive indicators among the aforementioned factors would result in a positive PERC score, suggesting a potentially higher risk of pulmonary embolism and thus necessitating further investigations and screenings for pulmonary embolism.
2. The relationship between D-dimer and PTE: D-dimer is an indicator that reflects the hypercoagulable state and fibrinolytic activity of the body. In the diagnosis of PTE, D-dimer demonstrates high sensitivity but relatively low specificity (6). Therefore, when utilizing D-dimer for PTE diagnosis, it is necessary to comprehensively consider other clinical indicators to make a more accurate judgment.
3. The role of electrocardiogram (ECG) in PTE diagnosis: Following the occurrence of PTE, ECG changes exhibit diversity, and some PTE patients may even present with completely normal ECGs (7). The lack of specificity in ECG alterations poses significant challenges for clinical differential diagnosis, which may subsequently lead to delays in the diagnosis and treatment of PTE.
4. The association between lower extremity venous ultrasonography and PTE: Examination of the deep veins of the lower extremities in PTE patients has revealed that a considerable proportion of them have deep venous thrombosis (DVT). The prevalence of DVT is significantly higher in elderly patients compared to non-elderly individuals, and there is a close relationship between DVT and PTE. Therefore, performing lower extremity venous ultrasonography in elderly patients suspected of having PTE holds important clinical significance (8). However, it is worth noting that even if the lower extremity venous ultrasonography appears normal, the possibility of pulmonary embolism cannot be completely ruled out.
5. Application of Wells Score in PTE Assessment: According to large-scale data, a lower Wells score indicates a lower likelihood of pulmonary embolism as perceived by clinicians (9, 10). However, with the deepening of research on pulmonary embolism, it has been found that many patients assessed as low-risk by the Wells score are still diagnosed with acute pulmonary embolism after undergoing pulmonary artery CTA examination. Therefore, when elderly patients present with symptoms such as hypoxemia or have underlying diseases such as chronic obstructive pulmonary disease, even if their Wells score is low, we still need to be highly vigilant about the possibility of acute pulmonary embolism.
6. The Role of Pulmonary Artery CTA in PTE Diagnosis: Currently, pulmonary artery CTA has become an important method for diagnosing pulmonary embolism (11, 12). However, clinical observations have revealed an overuse of pulmonary artery CTA, primarily driven by the concerns of treating physicians about missing acute pulmonary embolism. This overuse not only increases the economic burden on patients but also exposes them to potential risks of malignancy due to excessive ionizing radiation and adverse effects such as nephropathy and allergic reactions caused by contrast agents. Therefore, pulmonary artery CTA should primarily be used for the confirmation or exclusion of suspected pulmonary embolism patients and should not be employed as a routine examination tool.

After a thorough analysis of the clinical data presented in this case study, we postulate that the misdiagnosis of the patient may have been attributed to several factors. Firstly, the patient’s prominent clinical symptoms at the time of admission, including fever, chest pain, and hemoptysis, were highly similar to those of pneumonia and pleurisy, leading the treating physician to initially suspect a pulmonary infectious disease. Secondly, the patient exhibited several high-risk factors for PTE, such as prolonged sitting, bilateral lower extremity edema, bradycardia, and abnormally elevated D-dimer levels. However, due to the clinician’s lack of experience, PTE-related examinations were not promptly initiated, and these symptoms were erroneously attributed to the patient’s advanced age and hypercoagulable state. Thirdly, during the patient’s stay at the primary hospital, neither echocardiography nor chest CT revealed any changes indicative of pulmonary embolism in the pulmonary arteries or right heart system, lacking typical evidence for a diagnosis of pulmonary embolism, which contributed to its easy misdiagnosis in clinical practice. Fourthly, the limited availability of equipment at the primary hospital precluded the possibility of performing comprehensive pulmonary artery CTA examinations, which is also one of the reasons for missed and misdiagnoses of pulmonary embolism in such settings. Fifthly, despite presenting with typical initial symptoms of pulmonary embolism, the patient’s lack of attention to their own condition led to a delay in seeking medical attention until the onset of infectious complications, resulting in a diagnostic bias by the treating physician.

When encountering patients with high-risk factors for PTE but exhibiting non-typical PTE signs in clinical practice, if the patients subsequently develop pulmonary infection complications and fail to respond significantly to anti-infective therapy, we should reassess and consider conducting a screening for PTE. This approach aims to enhance the diagnostic rate of PTE, ensuring timely, accurate diagnosis and treatment for the patients. During the review of this case, we rigorously adhered to the epidemiological characteristics, predisposing factors, clinical manifestations, diagnostic criteria, and treatment protocols outlined in the guidelines (13). Through comprehensive evaluation, meticulous screening, and thorough related examinations, we ultimately confirmed the diagnosis of pulmonary embolism for the patient. This diagnosis was based on sufficient evidence, ensuring the accuracy and reliability of the diagnosis. In terms of treatment, we administered a reasonable therapeutic regimen tailored to the patient’s specific condition and conducted follow-up evaluations. The current prognosis of the patient is acceptable, further validating the effectiveness of our diagnostic and therapeutic strategies.

The clinical symptoms of pneumonia are diverse and include fever, cough, chest tightness, and chest pain. In severe cases, difficulty breathing and hemoptysis may also occur. These symptoms share certain similarities with the clinical manifestations of pulmonary embolism, leading to potential confusion in diagnosis and subsequent missed diagnoses of pulmonary embolism, which can negatively impact patient prognosis. Given this, the accurate diagnosis of pulmonary embolism poses significant challenges, requiring clinicians to continuously enhance their differential diagnosis skills in daily practice to ensure timely identification and treatment of this disease.

## Data availability statement

The original contributions presented in the study are included in the article/supplementary material, further inquiries can be directed to the corresponding author.

## Ethics statement

The studies involving humans were approved by the Ethics Committee of Ankang Central Hospital. The studies were conducted in accordance with the local legislation and institutional requirements. Written informed consent for participation was not required from the participants or the participants’ legal guardians/next of kin in accordance with the national legislation and institutional requirements. Written informed consent was obtained from the individual(s) for the publication of any potentially identifiable images or data included in this article.

## Author contributions

YD: Writing – original draft. JL: Writing – original draft. QH: Writing – original draft, Writing – review & editing.
